# Benign regulation of short-chain fatty acids: the underlying mechanism of the beneficial effects of manual acupuncture on cognitive ability and the intestinal mucosal barrier in APP/PS1 mice

**DOI:** 10.3389/fnins.2025.1509581

**Published:** 2025-02-04

**Authors:** Ning Ding, Xin Hao, Yue Zhang, Yanxiang Zhang, Zhigang Li

**Affiliations:** ^1^Department of Acupuncture, Guang’anmen Hospital, China Academy of Chinese Medical Sciences, Beijing, China; ^2^School of Acupuncture-Moxibustion and Tuina, Beijing University of Chinese Medicine, Beijing, China; ^3^Dalian Women and Children’s Medical Center (Group), Dalian, China

**Keywords:** manual acupuncture, Alzheimer’s disease, short chain fatty acids (SCFAs), FFAR3, intestinal mucosal barrier, NF-κB

## Abstract

**Background:**

Gut microbiota dysbiosis is closely related to the occurrence and progression of Alzheimer’s disease (AD). The destruction of the intestinal mucosal barrier caused by a decrease in short-chain fatty acids (SCFAs) plays a key role in gut microbiota dysbiosis-induced neuroinflammation in AD. Our previous research confirmed for the first time that manual acupuncture (MA) can benignly modulate gut microbiota dysbiosis, alleviating the destruction of the intestinal mucosal barrier. However, the regulatory effect of MA on SCFAs remains elusive, and the underlying mechanism by which MA improves intestinal mucosal barrier function requires elucidation.

**Methods:**

In the APP/PS1 manual acupuncture (Am) group, MA was applied at Baihui (GV20), Yintang (GV29), and Zusanli (ST36). Probiotics were delivered to the APP/PS1 probiotic (Ap) group. Alterations in spatial learning and memory, intestinal barrier function, SCFAs in feces and serum, the expression of FFAR3 and NF-κB, and inflammatory cytokines were evaluated in each group.

**Results:**

Compared with those in the C57BL/6 control (Cc) group, cognitive ability was significantly decreased, SCFAs and FFAR3 expression were obviously decreased, intestinal barrier integrity was drastically impaired, and the expression of NF-κB and the levels of intestinal IL-1β and TNF-*α* were increased in the APP/PS1 control (Ac) group. These changes were reversed by MA and probiotics.

**Conclusion:**

MA can significantly reduce intestinal inflammation and alleviate destruction of the intestinal mucosal barrier in APP/PS1 mice. SCFAs/FFAR3/NF-κB may be important targets through which MA benignly regulates intestinal mucosal barrier function.

## Introduction

In the past decade, gut microbiota dysbiosis has been shown to be closely related to the occurrence and progression of Alzheimer’s disease (AD) and has been confirmed as an early diagnostic biomarker and effective therapeutic target for AD (Zou et al., 2023). Gut microbiota dysbiosis-induced neuroinflammation is one of the core pathological mechanisms of AD (Choi et al., 2022), and disruption of the intestinal mucosal barrier plays a key role in the pathological cascade of AD induced by gut microbiota dysbiosis (Chidambaram et al., 2022). As one of the important initial pathological changes in AD, distinctive gut microbiota dysbiosis in AD disrupts the intestinal mucosal barrier, induces systemic inflammation, damages the blood–brain barrier (BBB), causes intestinal and brain leakage, leads to a neuroinflammatory response in the central nervous system, and ultimately impairs cognitive function (Choi et al., 2022; Wang, 2023). Benign regulation of the gut microbiota, such as probiotic supplementation and fecal transplantation, can alleviate neuroinflammatory reactions among individuals with AD and improve cognitive function, which is currently considered the most effective immune regulation target for the gut-brain axis (Yoshikawa et al., 2022).

As the main signaling molecules of the gut-brain axis, short-chain fatty acids (SCFAs) play important roles in regulating central neuroinflammation and intestinal barrier function (Sorboni et al., 2022; Alminen, 2023). SCFAs are saturated fatty acids with fewer than 6 carbon atoms, mainly propionate, butyrate, and acetate, which are mainly produced by the anaerobic digestion of dietary fiber by the gut microbiota. Both clinical and animal studies have confirmed that SCFAs-producing bacteria and SCFAs levels are significantly reduced in AD (Nandwana et al., 2022; Choi and Mook-Jung, 2023). A decrease in SCFAs can affect the expression of tight junction proteins in endothelial cells, leading to dysfunction of the intestinal mucosal barrier and BBB and inducing intestinal and brain leakage (Bicknell et al., 2023; Gong et al., 2023). More importantly, SCFAs, as inhibitors of HDACs and activators of GPCRs, can exert anti-inflammatory effects by inhibiting the ERK/JNK/NF-κB pathway (Zhong et al., 2021; Choi and Mook-Jung, 2023). Research has shown that SCFAs bind to FFAR3, inhibiting the NF-κB pathway and significantly reducing the expression of inflammatory cytokines such as IL-1β and TNF-*α* (Chen et al., 2022; Wasén et al., 2022). This action of SCFAs directly alleviates local intestinal inflammation and protects the function of the intestinal mucosal barrier. In addition, it causes changes in intestinal pH levels, which promote the proliferation of opportunistic pathogens (Marć et al., 2022), increase the levels of proinflammatory mediators such as lipopolysaccharides (LPS) and induce central invasion of proinflammatory mediators, ultimately exacerbating neuroinflammatory reactions and cognitive impairment (Faulin and Estadella, 2023; Krishaa et al., 2023). Additionally, SCFAs from the intestine can enter the central nervous system and directly regulate the functional homeostasis of microglia, which plays an important role in the regulation of central nervous system inflammation by the gut microbiota (Singh et al., 2022; Yadav et al., 2023).

Acupuncture is an effective complementary treatment for AD as it ameliorates cognitive function and enhances the quality-of-life, with advantages such as fewer adverse reactions, economic convenience, and good compliance (Ke et al., 2024; Lin et al., 2024). Our previous studies have shown that manual acupuncture (MA) improves spatial cognitive function in AD mice (Ding et al., 2019) and alleviates pathological markers such as Aβ deposition (Jiang et al., 2019). At the same time, MA increases cerebral blood flow in relevant brain regions by regulating the PLA_2_-AA pathway (Ding et al., 2019; Ding et al., 2020). In addition, our previous studies showed that MA downregulated the NLRP3/Caspase1 and TLR4/NF-κB pathways in microglia and alleviated microglial polarization imbalance and neuroinflammation (Ding et al., 2017; Lu et al., 2019). Regarding the intestinal mechanism of MA against AD, studies have reported that acupuncture can increase microbial diversity and improve AD symptoms by regulating the gut brain axis (Zou et al., 2024). The specific mechanism includes suppression of peripheral and central nervous system inflammation reactions through stimulation of the vagus nerve in the gut and enhancement of the immune response by metabolites of the gut microbiota. Our previous research confirmed for the first time that manual acupuncture (MA) can benignly modulate gut microbiota dysbiosis, effectively alleviating the destruction of the intestinal mucosal barrier in APP/PS1 mice, significantly increasing the expression of the intestinal tight junction proteins occludin and ZO-1 (Hao et al., 2022), and decreasing LPS loading and systemic inflammation (Zhang et al., 2022). In addition, we found that the gut microbiota may play an important role in the improvement of cognitive function and intestinal barrier function by MA (Hao et al., 2022; Zhang et al., 2022). However, the regulatory effect of MA on SCFAs remains elusive, and the underlying mechanism by which MA improves intestinal mucosal barrier function needs to be elucidated. This study aimed to investigate the regulatory effect of MA on SCFAs and determine the key intestinal molecules involved in MA regulation of intestinal mucosal barrier function.

## Materials and methods

### Experimental animals

Male APP/PS1 mice and male C57BL/6 mice were purchased from the Cavens Biogle Academy of Model Animal Research (Suzhou) (animal lot: SCXK-Su-2018-0002). Both types of mice weighed 30.0 ± 2.0 g and were 6 months old. The animals were housed in Experimental Animal Center of Beijing University of Chinese Medicine at a controlled temperature (24 ± 2°C) and under a 12-h dark/light cycle, with sterile drinking water and a standard pellet diet available ad libitum. All experimental procedures and animal welfare tests were approved by the Animal Ethics Committee of Guang’anmen Hospital (ethics number: IACUC-GAMH-2024-014-SQ).Animal Grouping and Intervention.

A total of 12 C57BL/6 mice were used as the control (Cc) group, and 36 APP/PS1 mice were divided into three groups (*n* = 12 per group): the APP/PS1 control (Ac) group, the APP/PS1 manual acupuncture (Am) group, and the APP/PS1 probiotics (Ap) group.

The mice in the Am group were immobilized in mouse bags. Disposable sterile acupuncture needles (0.25 mm × 13 mm) (Beijing Zhongyan Taihe Medicine Company, Ltd.) were used. MAs at Baihui (GV20), Yintang (GV29), and Zusanli (ST36) were applied for 20 min, with transverse puncturing at a depth of 3 mm (Baihui and Yintang) and perpendicular puncturing at a depth of 4 mm (Zusanli). The mice in the Ap group were administered probiotics at 8.7 × 10^8^ CFU/g/day (Beijing Zhongke Yikang Biotechnology Company, Ltd.) by oral gavage. The above treatments were administered once a day for 36 consecutive days, with no treatment of the Cc and Ac groups. The mice in the Cc, Ac, and Ap groups were immobilized for 20 min in the same manner as the mice in the Am group. The duration of MA and selection of the acupoints were based on findings from our previous studies (Hao et al., 2022; Zhang et al., 2022).

### Animal handling and sample collection

For the experimental procedures, 12 mice from each group underwent the Morris water maze (MWM) test between Days 31 and 35. On Day 36, six mice from each group were randomly selected for intestinal permeability assessment, followed by immunofluorescent (IF) staining of small intestinal tissues (ileum). Fresh feces and serum were collected from six mice per group were collected for high performance liquid chromatography–tandem mass spectrometry (UPLC–MS/MS) analysis, and small intestinal tissues (ileum) were subjected to western blotting (WB) and enzyme-linked immunosorbent assay (ELISA). The timeline of experimental design is presented in Supplementary Figure S1.

#### MWM

Mice in each group were subjected to the hidden platform trial and the probe trial. The MWM test we used in this study has been described previously (Tian et al., 2019). In the hidden platform trial, the platform was positioned in the middle of the SW quadrants. Each mouse was released from one of four start locations and had 60 s to search for the hidden platform. At the end of each trial, the mouse was placed on the platform or allowed to stay there for 10 s. 4 trials per day were performed for 5 consecutive days. The escape latency in the hidden platform trial was recorded for subsequent analysis.

The probe trial was conducted on the day after the completion of the hidden platform test. The platform was removed and each mouse was placed in the pool once for 60 s. The platform crossover number and swimming trace were recorded. The data was automatically collected by a video camera (TOTA-450d, Japan) fixed to the ceiling and connected to a video recorder with an automated tracking system (China Daheng Group, China).

#### Intestinal permeability assessment

The mice were given 4 kDa FITC-dextran (Sigma-Aldrich, St. Louis, MO, United States) by oral gavage (0.6 mg/g) as previously described (Cani et al., 2008). After 4 h, blood was taken from the eyeball and centrifuged. The concentrations of FITC were determined in 100 μL serum samples with an excitation wavelength of 485 nm and an emission wavelength of 535 nm.

#### IF staining

Small intestine tissues were fixed with paraformaldehyde and sectioned via a freezing microtome (CM1900, Leica Corporation, Germany). For IF staining, the sections were washed in 0.1 M PBS and blocked in 0.1 M PBS containing 10% normal donkey serum and 0.5% Triton X-100 for 30 min. The sections were treated with rabbit FFAR3 antibody (1:100, Abcam, United States) or rabbit NF-κB p65 antibody (1:100, Abcam, United States) and incubated overnight. After being washed, the sections were exposed to Alexa Fluor 488-conjugated donkey anti-rabbit IgG (1:100, Abcam, United States). Finally, the sections were washed with PBS and stained with DAPI (DAPI, Abcam, Netherlands). After washing with PBS, the sections were observed under a confocal laser scanning microscope (SP8, Leica, United States).

Identical exposure times and image settings were used for each experiment. For each sample, three fields of view were randomly selected for detection (Ding et al., 2020). The mean optical densities of FFAR3 and NF-κB p65 were analyzed via ImageJ.

#### UPLC-MS/MS

Approximately 300 mg of each fecal sample and 150 μL of each serum sample were precisely weighed, and 50% aqueous acetonitrile was added, followed by vortexing for 5 min to extract the SCFAs. After centrifugation, the clear supernatants were collected. SCFAs were measured as previously described (Han et al., 2015). The contents of SCFAs in the feces and serum were compared between the groups.

#### WB analysis

After homogenization and protein extraction, SDS–PAGE was performed with an 8% separating gel and a 5% stacking gel, and the proteins were subsequently transferred to a PVDF membrane. The membranes were subsequently incubated with an anti-NF-κB p65 antibody (1:500, Abcam, United States), an anti-FFAR3 antibody (1:500, Abcam, United States), and an anti-*β*-actin antibody (1:500, Bioss, United States). Secondary antibodies conjugated to horseradish peroxidase (HRP) were added for 1 h, and the proteins were detected via an enhanced chemiluminescence (ECL) luminescent solution. All the bands were normalized to their corresponding β-actin expression levels for the appropriate evaluation of protein expression.

#### ELISA analysis

After homogenization of the small intestine tissues, the supernatant was collected. The concentrations of IL-1β and TNF-*α* in the intestine were determined via ELISA kits (Oubei Biotechnological Co. Ltd., Beijing).

### Statistical analysis

The statistical analysis was performed via SPSS software, version 17.0 (SPSS, Inc., Chicago, IL, United States), and the data are expressed as the means ± standard errors of the means. Two-way ANOVA with repeated measures was used to analyze group differences in escape latency. One-way ANOVA followed by the least significant difference (LSD) multiple-range test was used to analyze group differences. For non-normally distributed data or for data with heterogeneous variance, a Kruskal–Wallis test was used. Statistical significance was set to *p* < 0.05, and high statistical significance was set to *p* < 0.01.

## Results

### Effect of MA on spatial learning and memory

**Figure 1:**
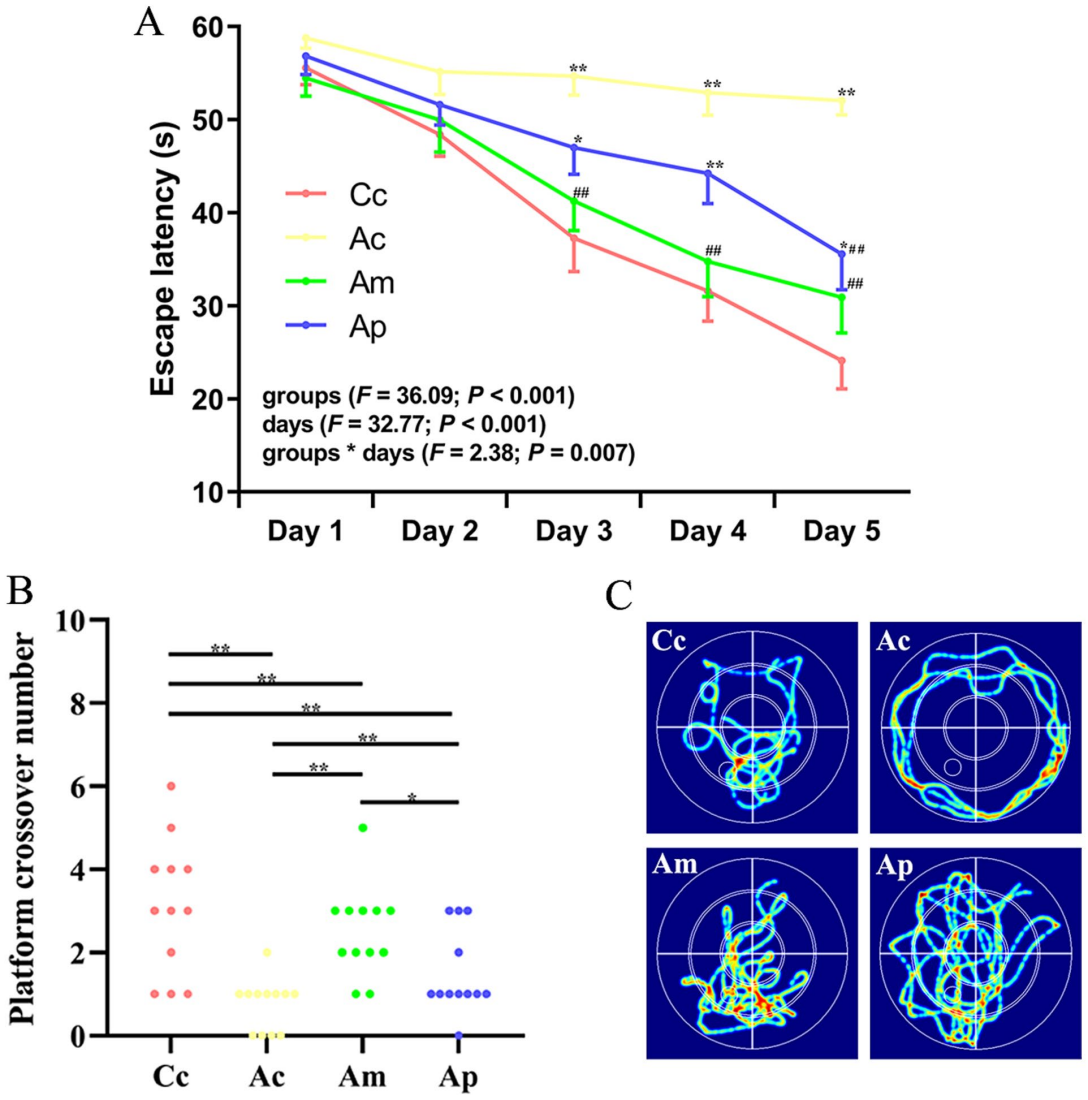
Results of the hidden platform and probe trials in each group (*n* = 12, mean ± SEM). **(A)** Comparison of the escape latency of all groups on the hidden platform. Two-way ANOVA with repeated measures was used. The LSD-t results are presented in Supplementary Table S1. **(B)** Comparison of the platform crossover numbers of all groups. Platform crossover numbers were analyzed via the Kruskal–Wallis test. **(C)** Swimming trajectories in all groups. ^**^p < 0.01, ^*^p < 0.05 compared with the Cc group. ^##^*p* < 0.01, ^#^*p* < 0.05 compared with the Ac group.

The results of the hidden platform and probe trials in the MWM are presented in Figure 1. In the hidden platform trial, the escape latency of the Cc, Am and Ap groups decreased gradually, but the Ac group maintained a long latency. There were no significant group differences in escape latency on Days 1–2. Compared with that in the Cc group, the escape latency in the Ac group significantly increased from Days 3–5 (*p* < 0.01). The escape latency of the Ap group was notably greater than that of the Cc group from Days 3–5 (*p* < 0.01 or *p* < 0.05). Compared with that in the Ac group, the escape latency in the Am and Ap groups substantially decreased from Days 3–5 and Day 5, respectively (*p* < 0.01).

The platform crossover number in the Ac and Ap groups was significantly lower than that in the Cc and Am groups (*p* < 0.01 or *p* < 0.05). The platform crossover numbers in the Am and Ap groups were greater than those in the Ac group (*p* < 0.01), whereas the platform crossover frequency was still lower than that in the Cc group (*p* < 0.01). For the search strategy, the swimming activity was mostly concentrated in the targeted quadrant in the Cc, Am, and Ap groups. In contrast, the swimming activity in the Ac group was mostly random. A comparison of the swimming speeds of all the groups in the behavioral tests is presented in Supplementary Figure S2, and no significant group differences were observed.

### Effect of MA on SCFAs in the feces and serum

**Figure 2:**
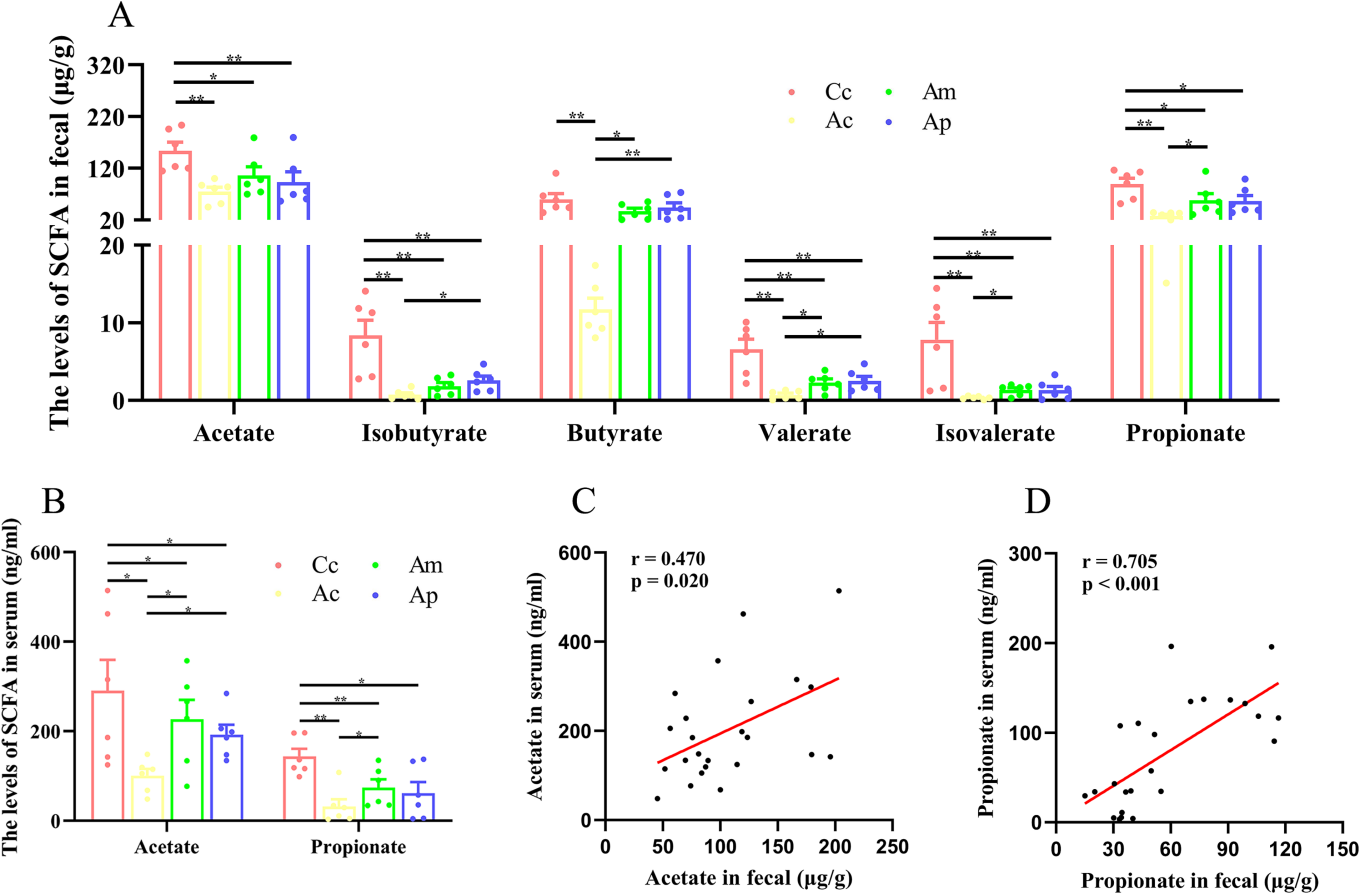
SCFAs results for each group (*n* = 6, mean ± SEM). **(A)** Comparison of SCFAs levels in the feces of all the groups. One-way ANOVA followed by the least significant difference (LSD) multiple-range test was used, except for the comparisons of isobutyrate, valerate, and isovalerate, which were analyzed via the Kruskal-Wallis test. **(B)** Comparison of the SCFAs in the serum of all the groups. Acetate and propionate were analyzed by the Kruskal-Wallis test. **(C,D)** Pearson correlation analysis of acetate and propionate concentrations in feces and serum. The LSD-t test and chi-square test results are presented in Supplementary Tables S2, S3.

The effects of MA on SCFAs in the feces and serum are presented in Figure 2. In the feces, the contents of acetate, isobutyrate, valerate, isovalerate, and propionate in the Ac, Am, and Ap groups were significantly lower than those in the Cc group (*p* < 0.01 or *p* < 0.05). The content of butyrate in the Ac group was significantly lower than that in the Cc group (*p* < 0.01). Compared with those in the Ac groups, the contents of isobutyrate, butyrate, and valerate in the Ap group and of butyrate, valerate, isovalerate, and propionate in the Am group were markedly greater (*p* < 0.01 or *p* < 0.05).

In the serum, the contents of acetate and propionate in the Ac, Am, and Ap groups were significantly lower than those in the Cc group (*p* < 0.01 or *p* < 0.05). Compared with those in the Ac groups, the contents of acetate and propionate in the Am group and propionate in the Ap group were notably greater (*p* < 0.05). Pearson correlation analysis revealed a positive correlation between acetate in the feces and serum of the mice (*r* = 0.470, *p* = 0.020), and there was a positive correlation between propionate in the feces and serum of the mice (*r* = 0.705, *p* < 0.001).

### Effect of MA on the expression of FFAR3 and NF-κB in the intestine

**Figure 3:**
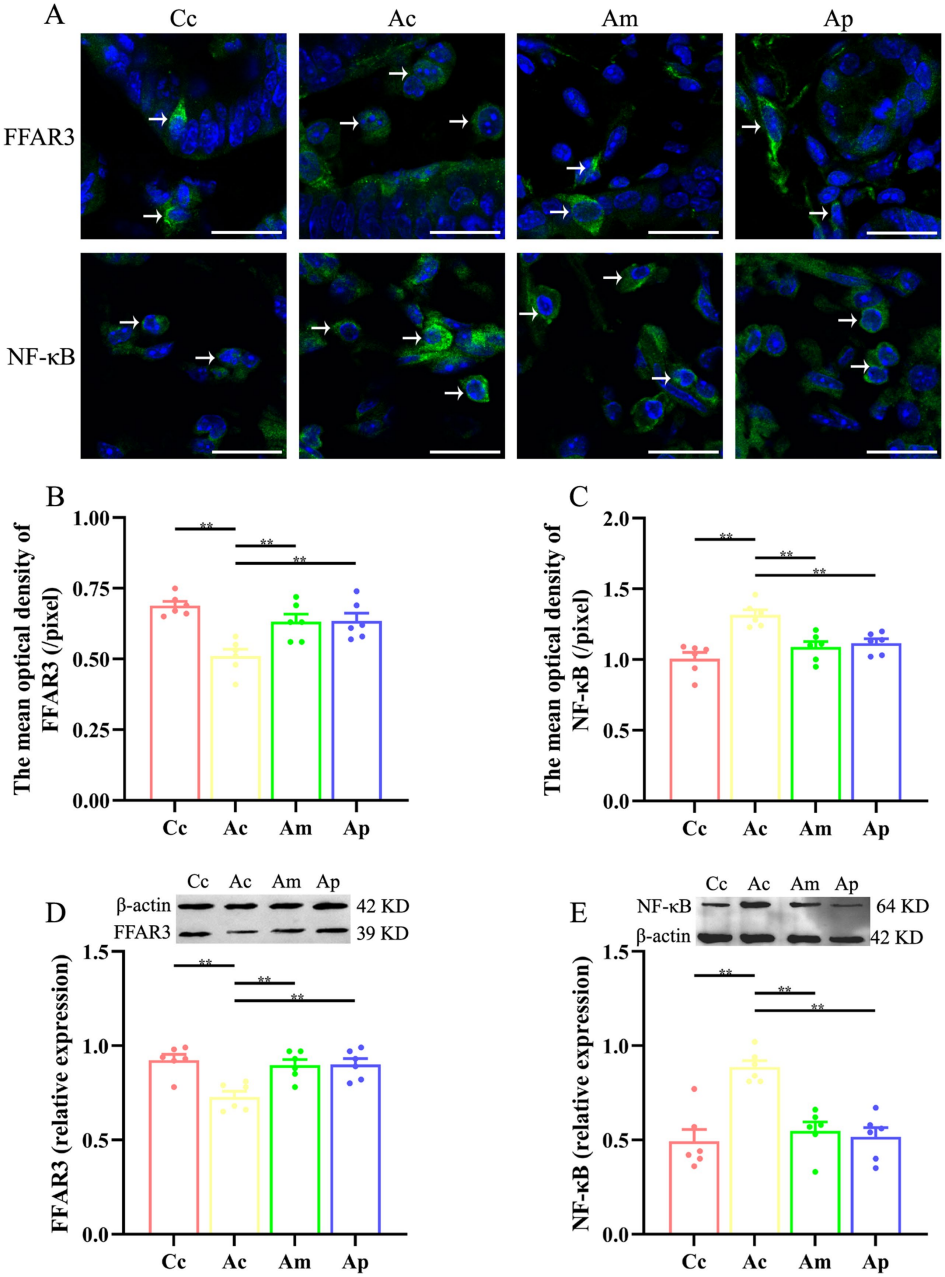
Results of FFAR3 and NF-κB expression in each group (*n* = 6, mean ± SEM). **(A)** Representative images of immunofluorescence staining of FFAR3 and NF-κB in each group. The positively stained cells are indicated by white arrows. The scale bar is 20 μm. **(B,C)** Comparison of the mean optical density of FFAR3 and NF-κB in each group. **(D,E)** Comparison of the relative expression of FFAR3 and NF-κB in each group. One-way ANOVA followed by the least significant difference (LSD) multiple range test was used. The LSD-t results and original data are presented in Supplementary Tables S3, S4; Supplementary Figures S3, S4.

The effects of MA on the expression of FFAR3 and NF-κB in the intestine are presented in Figure 3. FFAR3 and NF-κB are found mainly in the lamina propria of the intestine and are ovoid in shape. The fluorescence intensity of FFAR3 in the Ac group was lower than that in the Cc group, whereas the fluorescence intensity of NF-κB in the Ac group was greater than that in the Cc group. Compared with that of the Ac group, the fluorescence intensity of FFAR3 was greater in the Am and Ap groups. The fluorescence intensity of NF-κB in the Am and Ap groups was lower than that in the Ac group.

The mean optical density and relative expression of FFAR3 in the Ac group were significantly lower than those in the Cc group (*p* < 0.01). Compared with those in the Ac group, the mean optical density and relative expression of FFAR3 in the Am and Ap groups were markedly greater (*p* < 0.01). The mean optical density and relative expression of NF-κB in the Ac group were significantly greater than those in the Cc group (*p* < 0.01). The mean optical density and relative expression of NF-κB in the Am and Ap groups decreased markedly (*p* < 0.01).

Effect of MA on the intestinal mucosal barrier and inflammatory cytokines.

**Figure 4:**
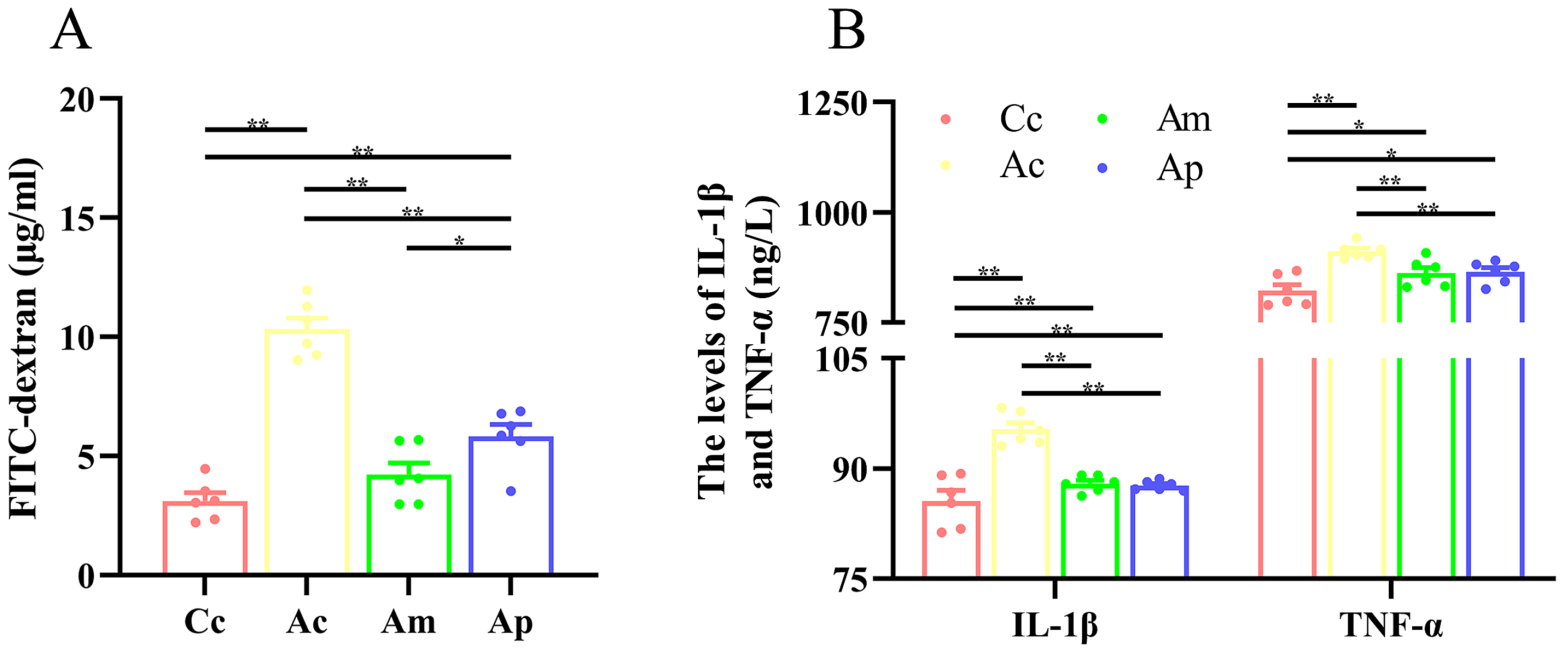
Results of the intestinal mucosal permeability and inflammatory cytokines in each group (*n* = 6, mean ± SEM). **(A)** Comparison of FITC-dextran in all groups. One-way ANOVA was used. **(B)** Comparison of the IL-1β and TNF-*α* levels among all the groups. The Kruskal-Wallis test was used. The LSD-t test and chi-square test results are presented in Supplementary Tables S2, S3.

The effects of MA on the intestinal mucosal barrier and inflammatory cytokines are presented in Figure 4. The levels of FITC-dextran, IL-1β, and TNF-*α* in the Ac group were significantly greater than those in the Cc group (*p* < 0.01). Markedly lower FITC-dextran, IL-1β, and TNF-α levels were detected in the Am and Ap groups than in the Ac group (*p* < 0.01). FITC-dextran in the Ap group was greater than that in the Cc and Am groups (*p* < 0.01 or *p* < 0.05). IL-1β and TNF-α levels were greater in the Am and Ac groups than in the Cc group (*p* < 0.01 or *p* < 0.05).

## Discussion

In the hidden platform trial of the Morris water maze, the decreasing trend of the escape latency in the Am group was more apparent and rapid than that in the Ap group. The escape latency of the Am group was significantly lower than that of the Ac group beginning on Day 3, whereas the escape latency of the Ap group was significantly lower than that of the Ac group beginning on Day 5. There was no significant difference in escape latency between the Am and Cc groups, but the escape latency of the Ap group remained significantly greater than that of the Cc group from Days 3 to 5. The results indicated that MA has characteristics of improved efficiency and rapidity in terms of spatial learning ability, which is to some extent superior to probiotics. This trend was also reflected in the probe trial; although both the Am and Ap groups showed significant improvement compared with the Ac group, the platform crossover number in the Am group was significantly greater than that in the Ap group, reflecting the advantage of MA in spatial memory. The results of this study are consistent with our previous findings (Ding et al., 2019), indicating a specific advantage of MA in improving spatial cognitive ability. The MA intervention protocol used in this study can improve the cognitive function of APP/PS1 mice and provide useful references for similar research. The results also suggest that probiotics have beneficial regulatory effects on cognitive function, which is consistent with relevant research (Zhu et al., 2023). We inferred that the advantages of MA in improving cognitive function may be related to its multitarget characteristics. Our previous studies revealed that the regulatory effect of MA on AD pathological processes is not limited to regulating intestinal microbiota dysbiosis; MA can also alleviate neuroinflammation (Ding et al., 2017; Jiang et al., 2018; Lu et al., 2019), improve cerebral blood flow (Ding et al., 2019), enhance brain metabolism (Jiang et al., 2015), and reduce the expression of pathological markers (Jiang et al., 2019). Subsequent studies should explore the effects of MA on cognitive function in APP/PS1 mice of different ages and further explore the time and effect patterns of MA in improving cognitive function.

Both the SCFAs content in the feces and the serum was significantly reduced in APP/PS1 mice, which is consistent with relevant research findings (Nandwana et al., 2022), indicating that the pathological changes in SCFAs in 6-month-old APP/PS1 mice are associated with a decrease in the number of SCFAs in AD and can serve as effective research objects. These results indicate that MA and probiotics have clear regulatory effects on SCFAs in feces and serum and that there is a positive correlation between the levels of acetate and propionate in serum and feces. This result confirms, for the first time, that MA can effectively regulate AD pathological changes in intestinal and serum SCFAs disorders, which corresponds to our previous findings of the benign regulation of MA on gut microbiota dysbiosis (Hao et al., 2022; Zhang et al., 2022). SCFAs maybe the key effector molecules in MA regulation of gut microbiota disorders and may possibly play an important role in the regulation of cognitive function by MA. Given that SCFAs play important roles in regulating immune cell function and protecting barrier function in AD (Qian et al., 2022), these results suggest that the benign regulation of SCFAs may be an important link in MA to improve cognitive function. In regulating the SCFAs, propionate, butyrate, and acetate contents to the greatest extent, MA can upregulate the levels of propionate, butyrate, and acetate. Among them, propionate and acetate had statistically significant effects on feces and serum, whereas butyrate had a statistically significant effect on serum, which was superior to that of the Ap group. Additionally, the results showed that MA and probiotics have relative specificity in regulating SCFAs. In the feces, MA mainly reflects the regulation of isobutyrate and propionate, whereas probiotics predominantly regulate isobutyrate and butyrate, with both demonstrating significant regulatory effects on valerate. The multi-target regulatory trends of SCFA observed in this study are consistent with the findings of related research (Tian et al., 2024a). Previous studies have demonstrated that SCFAs are produced mainly by Firmicutes and Bacteroidetes (Borbolis et al., 2023), and these bacterial phyla are key targets for interventions aiming to modulate SCFA production (Tian et al., 2022; Tian et al., 2024b). Our previous studies also revealed that MA can significantly increase the abundance of Bacteroidetes in APP/PS1 mice and positively regulate the level of Firmicutes (Hao et al., 2022), which may be a potential mechanism by which MA can regulate SCFAs in a benign manner. Additionally, our previous studies revealed a certain degree of separation between MA and probiotics in the *β* diversity analysis of the gut microbiota (Hao et al., 2022), suggesting that MA and probiotics have different regulatory effects on the gut microbiota. Therefore, the different targeted microbiota are considered one of the main reasons for the heterogeneity in regulating SCFAs between MA and probiotics. Combined with correlation analysis with 16S rRNA sequencing, the key microbiota targeted by MA in the regulation of SCFAs should be identified, and the intervention effect of MA on brain SCFAs should be explored to further clarify the key SCFAs involved in MA regulation.

This study found that MA can significantly reduce the levels of IL-1β and TNF-*α* in the intestine and decrease FITC levels, which is the same effect as probiotics. The results confirmed that MA improved intestinal inflammation levels and protected intestinal mucosal permeability, which was consistent with our previous findings (Hao et al., 2022), indicating the anti-inflammatory effect of MA at the intestinal level. Research has shown that SCFAs are important regulatory factors that mediate NF-κB signaling and are key regulators of intestinal permeability (Li et al., 2021; Krishaa et al., 2023). To the best of our knowledge, this study is the first to report that MA can significantly reverse the decrease in FFAR3 expression in the intestine of APP/PS1 mice and inhibit downstream NF-κB expression, suggesting that SCFAs/FFAR3/NF-κB may be an important target for the benign regulation of intestinal mucosal barrier function in MA, further revealing the potential mechanism by which MA reduces intestinal inflammation. On the basis of our existing research (Hao et al., 2022; Zhang et al., 2022), we inferred that MA may increase SCFAs levels by regulating gut microbiota dysbiosis, alleviate intestinal inflammation levels through the FFAR3/NF-κB pathway, and ultimately protect the intestinal mucosal barrier. Research has shown that immune cells, intestinal endocrine cells, and other cells all express SCFAs receptors, which can promote GLP-1/PSY secretion through GPR41/43 and inhibit inflammatory responses through the GPR109A and HDAC pathways (Lee et al., 2021; Mirzaei et al., 2021) involved in decreasing intestinal anti-inflammatory effects caused by MA need further exploration. In addition, although there was no significant difference in the regulatory effects of MA and probiotics on FFAR3 and NF-κB, the benign regulatory effect of MA on intestinal mucosal barrier permeability was greater than that of probiotics, which was reflected by the statistically significant difference in FITC-dextran between the Am and Ap groups, indicating that the regulation of intestinal mucosal barrier function by MA may not be limited to anti-inflammatory mechanisms. Further exploration of the structural basis and molecular mechanisms of MA in regulating intestinal mucosal permeability is needed in future studies.

This study has some noteworthy limitations. First, while there are currently no published reports on the effects of long-term gavage-related stress on microbiota and cognition, the possibility of interference from related procedures cannot be excluded. In future studies, incorporating supplementation through feed or water may help mitigate some of these potential effects. In addition, germ-free mice or fecal microbiota transplantation could be used in subsequent studies to further validate the role of microbiota in MA regulation of the intestinal mucosal barrier. Second, the specificity of MA and probiotics in regulating SCFA, as observed in this study, requires further validation by increasing the sample size. Also, the use of multi-omics approaches, including 16S rRNA sequencing and metabolomic analysis, could provide a deeper understanding of the regulatory effects of MA on SCFA and its target microbiota. In addition, existing studies suggest that the regulatory mechanism of SCFA on the NF-κB pathway include not only the upregulation of FFAR3 expression and inhibition of the ERK pathway but also the activation of the MAPK pathway via FFAR2 (Luo et al., 2024). Further research should elucidate the roles of SCFAs and their pathways in MA-mediated regulation of intestinal inflammation.

## Conclusion

This result confirms, for the first time, that MA can regulate AD pathological changes in intestinal and serum SCFAs disorders, which may play an important role in the regulation of cognitive function by MA. SCFAs maybe the key effector molecules in MA-mediated regulation of gut microbiota disorders. Additionally, MA significantly reversed the decrease in FFAR3 expression in the intestine of APP/PS1 mice and inhibited downstream NF-κB expression, suggesting that SCFAs/FFAR3/NF-κB may be important targets through which MA benignly regulates intestinal mucosal barrier function.

## Data Availability

The original contributions presented in the study are included in the article/Supplementary material, further inquiries can be directed to the corresponding author.
