# Supplementary material for: Benign regulation of short-chain fatty acids: the underlying mechanism of the beneficial effects of manual acupuncture on cognitive ability and the intestinal mucosal barrier in APP/PS1 mice
Source: Front Neurosci. 2025 Feb 4;19:1509581. doi: 10.3389/fnins.2025.1509581 (PMC11832542; doi:10.3389/fnins.2025.1509581)
Supplement: Supplementary file 1 [file Table_1.doc]

**SUPPLEMENTARY TABLE 1 | The LSD-t of escape latency in hidden platform trial (t, P).**

| **Groups** | **Day 3** | **Day 4** | **Day 5** |
| --- | --- | --- | --- |
| Ac | Cc (-4.14; < 0.001) | Cc (-4.68; < 0.001) | Cc (-6.17; < 0.001) |
| Am | Ac (3.19; 0.003) | Ac (3.98; < 0.001) | Ac (6.17; < 0.001) |
| Ap | Cc (-2.31; 0.025) | Cc (-2.78; 0.008) | Cc (-2.52; 0.015)  Ac (3.64; 0.001) |

**SUPPLEMENTARY TABLE 2 | The Chi-Square (Chi-Square, P).**

| **Platform crossover number** | **Ac** | **Am** | **Ap** |
| --- | --- | --- | --- |
| Cc | (12.09; < 0.001) | (17.24; < 0.001) | (20.43; < 0.001) |
| Ac |  | (13.39; < 0.001) | (14.79; 0.001) |
| Am |  |  | (4.65; 0.031) |
| **Acetate in serum** |  |  |  |
| Cc | (6.56; 0.010) | (7.73; 0.021) | (9.45; 0.024) |
| Ac |  | (4.33; 0.037) | (7.45; 0.024) |
| **Propionate in serum** |  |  |  |
| Cc | (7.41; 0.006) | (10.89; 0.004) | (10.57; 0.014) |
| Ac |  | (4.33; 0.037) |  |
| **Isobutyrate in feces** |  |  |  |
| Cc | (8.31; 0.004) | (10.68; 0.003) | (14.65; 0.002) |
| Ac |  |  | (7.63; 0.022) |
| **Valerate in feces** |  |  |  |
| Cc | (8.31; 0.004) | (12.36; 0.002) | (15.42; 0.001) |
| Ac |  | (5.77; 0.016) | (8.99; 0.011) |
| **Isovalerate in feces** |  |  |  |
| Cc | (8.31; 0.004) | (10.67; 0.005) | (11.53; 0.009) |
| Ac |  | (5.03; 0.025) |  |
| **IL-1β** |  |  |  |
| Cc | (8.31; 0.004) | (11.34; 0.003) | (13.76; 0.003) |
| Ac |  | (8.34; 0.004) | (11.48; 0.003) |

**SUPPLEMENTARY TABLE 3 | The LSD-t (t, P).**

| **Acetate in feces** | **Ac** | **Am** | **Ap** |
| --- | --- | --- | --- |
| Cc | (3.53; 0.002) | (2.13; 0.046) | (2.70; 0.014) |
| **Butyrate in feces** |  |  |  |
| Cc | (4.33; < 0.001) |  |  |
| Ac |  | (-2.26; 0.035) | (-2.93; 0.008) |
| **Propionate in feces** |  |  |  |
| Cc | (4.33; < 0.001) | (2.20; 0.040) | (2.31; 0.031) |
| Ac |  | (2.34; 0.046) |  |
| **Relative expression of FFAR3** |  |  |  |
| Cc | (4.58; < 0.001) |  |  |
| Ac |  | (-3.97; 0.001) | (-4.03; 0.001) |
| **Relative expression of NF-κB** |  |  |  |
| Cc | (-5.66; < 0.001) |  |  |
| Ac |  | (4.85; < 0.001) | (5.32; < 0.001) |
| **The mean optical density of FFAR3** |  |  |  |
| Cc | (5.34; < 0.001) |  |  |
| Ac |  | (-3.68; 0.001) | (-3.71; 0.001) |
| **The mean optical density of NF-κB** |  |  |  |
| Cc | (-5.81; < 0.001) |  |  |
| Ac |  | (4.24; < 0.001) | (3.73; 0.001) |
| **FITC-dextran** |  |  |  |
| Cc | (-11.13; < 0.001) |  | (-4.18; < 0.001) |
| Ac |  | (9.43; < 0.001) | (6.95; < 0.001) |
| Am |  |  | (-2.48; 0.022) |
| **TNF-α** |  |  |  |
| Cc | (-5.63; < 0.001) | (-2.52; 0.020) | (-2.66; 0.015) |
| Ac |  | (3.11; 0.006) | (2.97; 0.008) |

**SUPPLEMENTARY TABLE 4 | Results of FFAR3 and NF-κB expression in each group (n=6, mean ± SEM).**

| **Groups** | **Cc** | **Ac** | **Am** | **Ap** |
| --- | --- | --- | --- | --- |
| The mean optical density of FFAR3 | 0.69±0.01 | 0.51±0.02 | 0.63±0.03 | 0.63±0.03 |
| The mean optical density of NF-κB | 1.01±0.04 | 1.32±0.04 | 1.09±0.04 | 1.12±0.03 |
| The relative expression of FFAR3 | 0.92±0.03 | 0.73±0.03 | 0.90±0.03 | 0.90±0.03 |
| The relative expression of NF-κB | 0.49±0.06 | 0.89±0.03 | 0.55±0.05 | 0.52±0.05 |
