# Supplementary figures and images for: Benign regulation of short-chain fatty acids: the underlying mechanism of the beneficial effects of manual acupuncture on cognitive ability and the intestinal mucosal barrier in APP/PS1 mice
Source: Front Neurosci. 2025 Feb 4;19:1509581. doi: 10.3389/fnins.2025.1509581 (PMC11832542; doi:10.3389/fnins.2025.1509581)

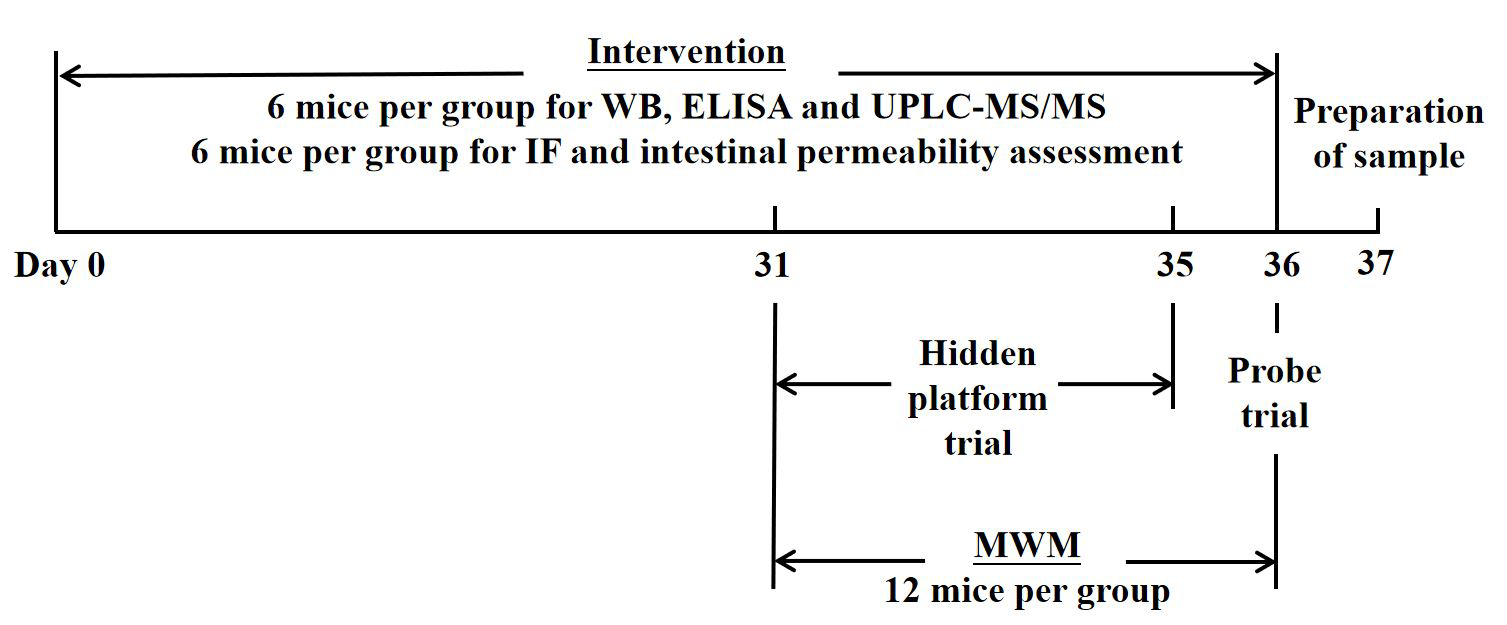

Supplement: Supplementary file 2 [file Image_1.tif]

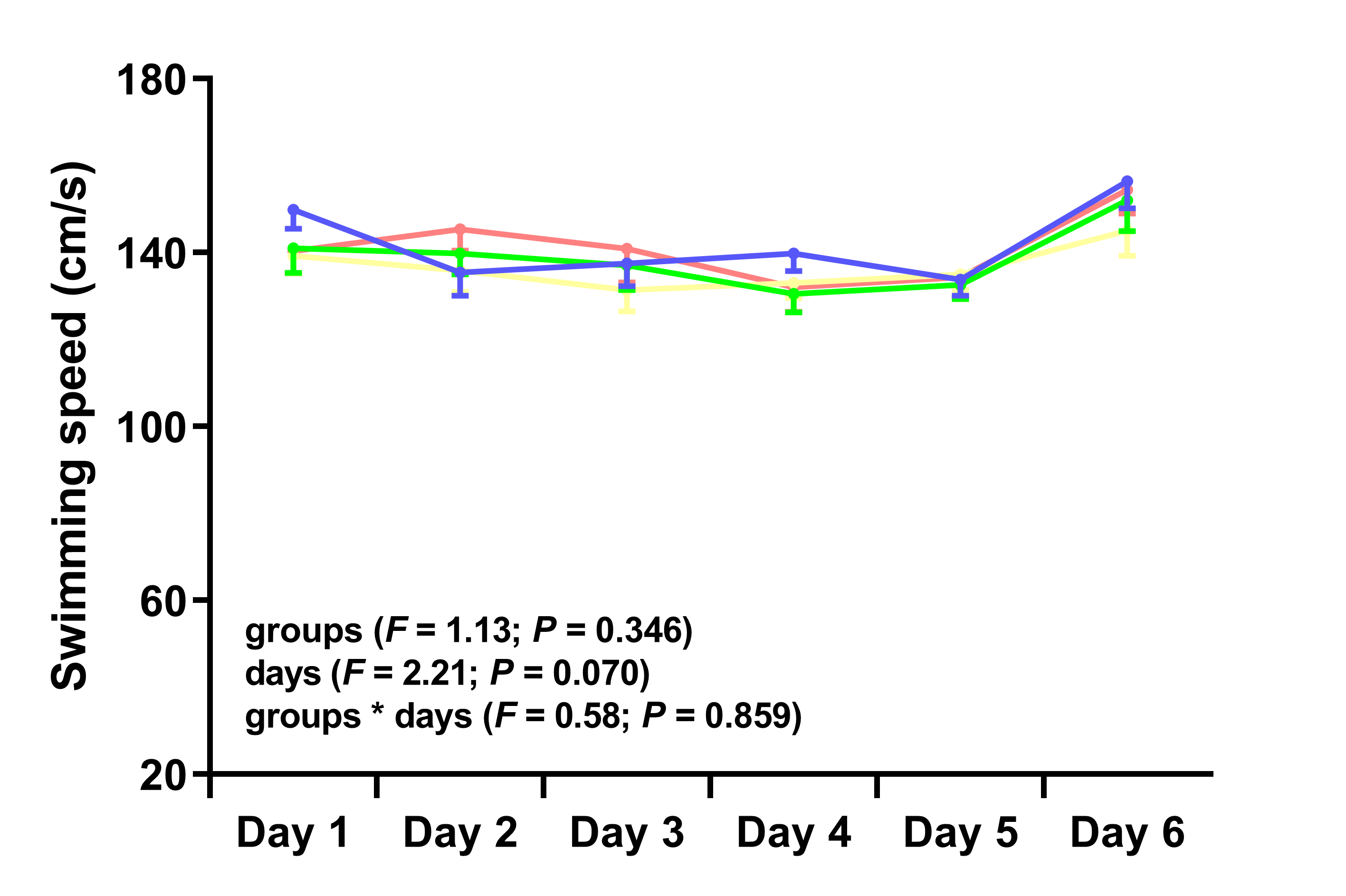

Supplement: Supplementary file 3 [file Image_2.tif]

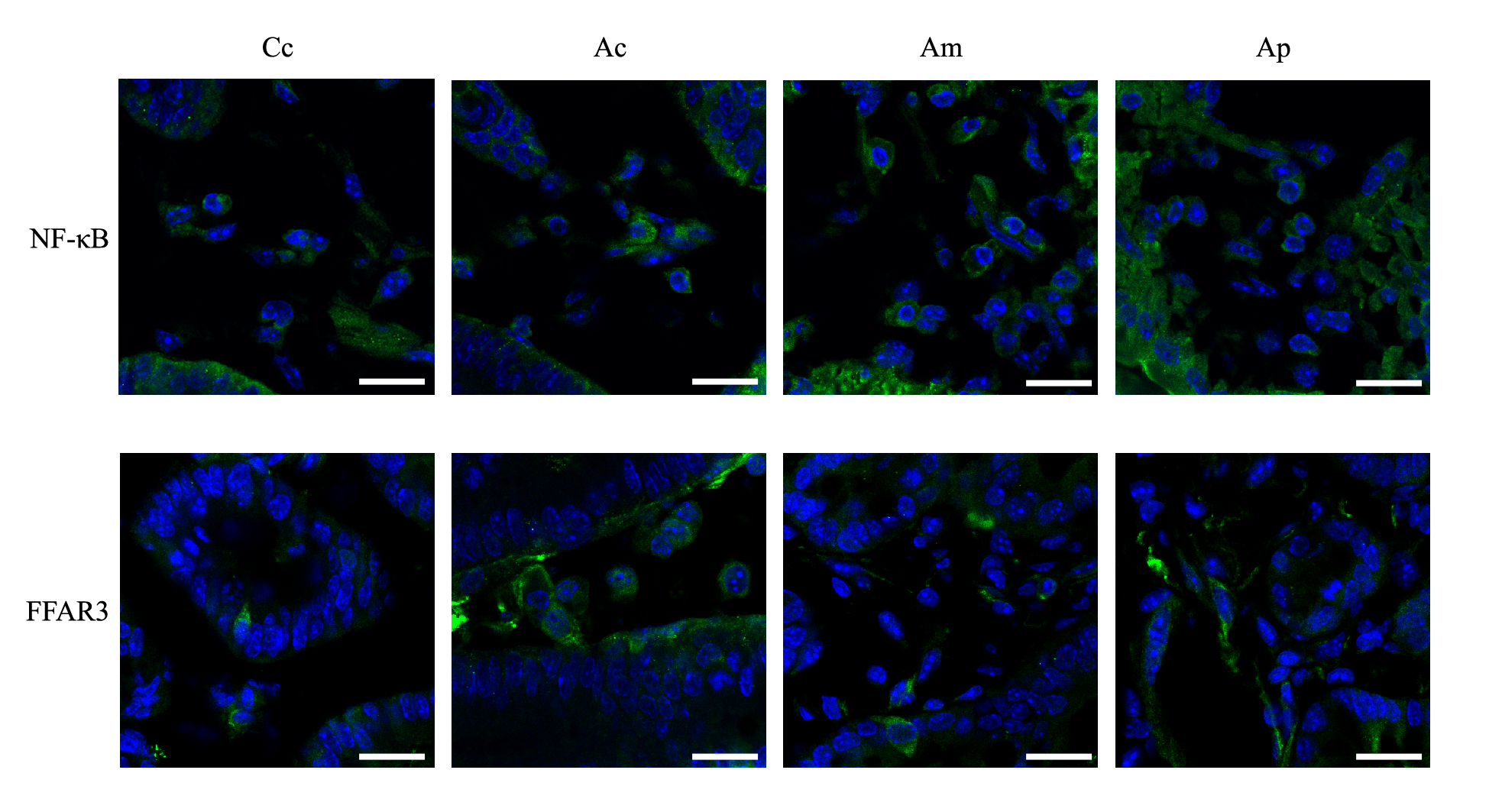

Supplement: Supplementary file 4 [file Image_3.tif]

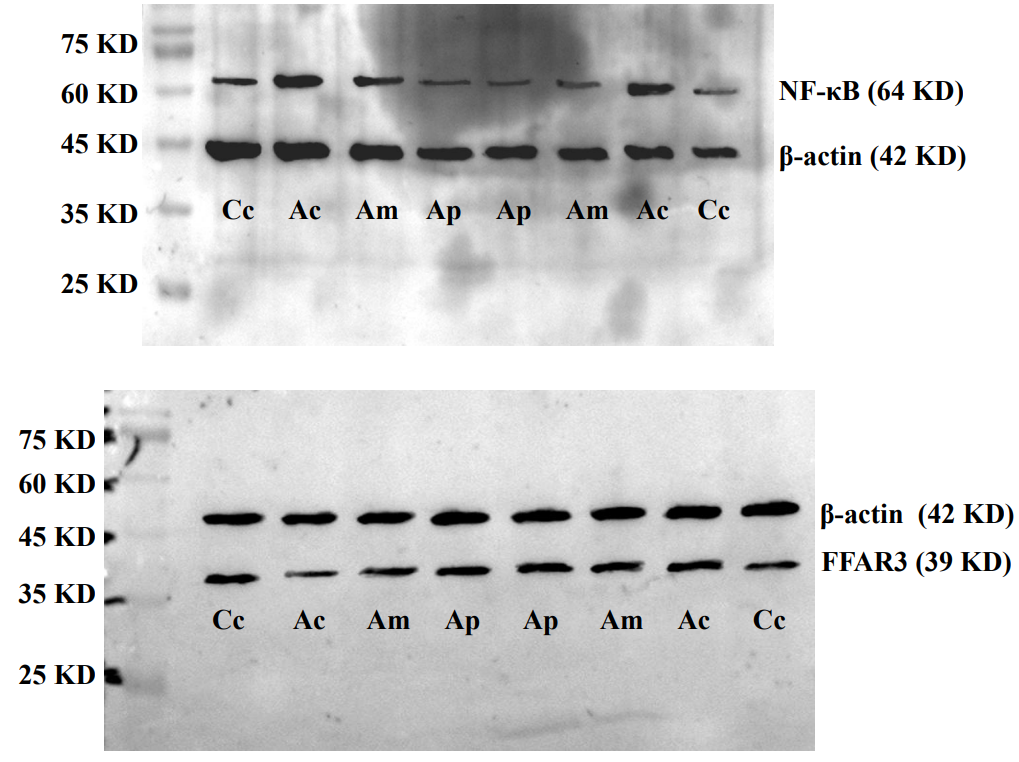

Supplement: Supplementary file 5 [file Image_4.png]
